# Supplementary material for: Brain-specific inhibition of mTORC1 eliminates side effects resulting from mTORC1 blockade in the periphery and reduces alcohol intake in mice
Source: Nat Commun. 2021 Jul 27;12:4407. doi: 10.1038/s41467-021-24567-x (PMC8316332; doi:10.1038/s41467-021-24567-x)
Supplement: Supplementary file 1 — Supplementary information [file 41467_2021_24567_MOESM1_ESM.pdf]

Supplementary information:

Brain-Specific Inhibition of mTORC1 Eliminates Side Effects Resulting from mTORC1 Blockade in the Periphery and Reduces Alcohol Intake in Mice

Yann Ehinger[1], Ziyang Zhang[2], Khanhky Phamluong[1], Drishti Soneja[1], Kevan M. Shokat[2], Dorit Ron[1]*

[1] Department of Neurology, University of California San Francisco

[2] Department of Cellular and Molecular Pharmacology and Howard Hughes Medical Institute, University of California, San Francisco, California

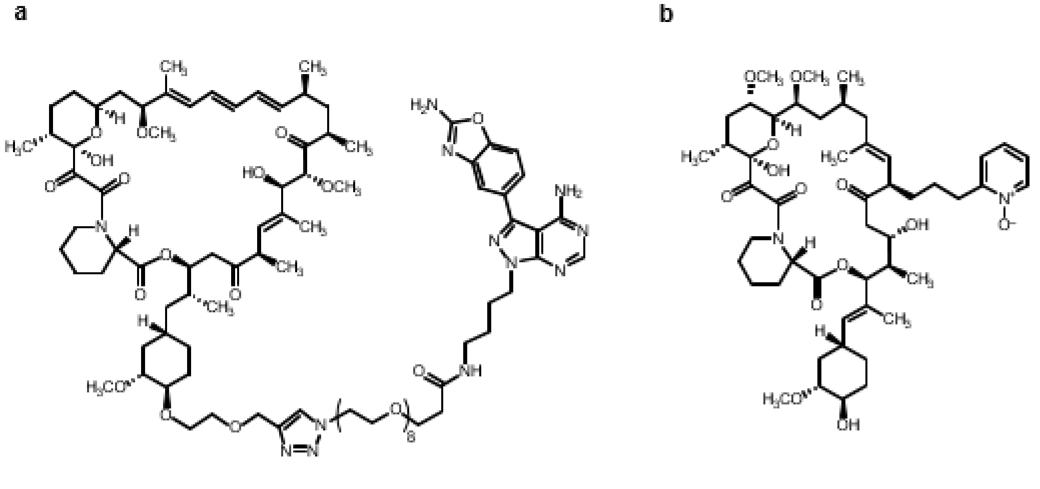

RapaLink-1 RapaBlock

Supplementary Fig. 1. Structure of RapaLink-1 and RapaBlock. RapaLink-1 is a cell and brain permeable small molecule consisting of the FKBP-12 binding molecule rapamycin, and an mTOR kinase inhibitor which are covalently linked[1]. RapaBlock is a brain impermeable, FKBP-12 binding molecule[2].

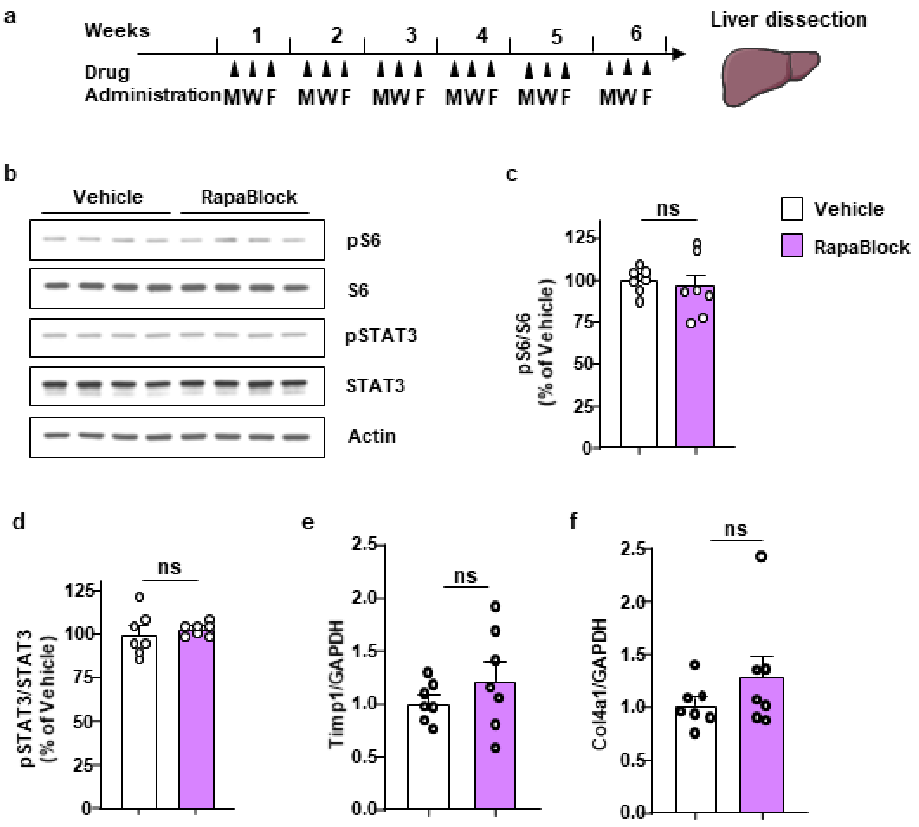

Supplementary Fig. 2. Chronic administration of RapaBlock does not alter mTORC1 activity and liver toxicity. (a) Timeline of experiments. Mice were treated with vehicle (white) or RapaBlock (40mg/kg, pink) 3 times a week for 6 weeks. (b-c) The liver was dissected 24 hours after the last drug administration, and S6 and STAT3 phosphorylation were measured. (b) Representative images of pS6, total S6 (top panels), pSTAT3, total STAT3 (middle panels) and actin (bottom panel). (c) RapaBlock does not affect mTORC1 activity in the liver (Two tailed unpaired t test: t = 0.5774, p = 0.5743, r^2 = 0.02703). (d) Administration of RapaBlock does not affect STAT3 phosphorylation level in the liver (Two tailed unpaired t test: t = 0.6176, p = 0.5484, r^2 = 0.0308). (e,f) Chronic RapaBlock treatment does not affect the level of the fibrogenic markers Timp1 (Two tailed unpaired t test: t = 1.101, p = 0.2925, r^2 = 0.09174), and Col4a1 (Two tailed unpaired t test: t = 1.218, p = 0.2465, r^2 = 0.1101). Data are presented as individual data points and mean ± SEM. Significance was determined using Two tailed unpaired t test. n=7 per condition. ns = non-significant.

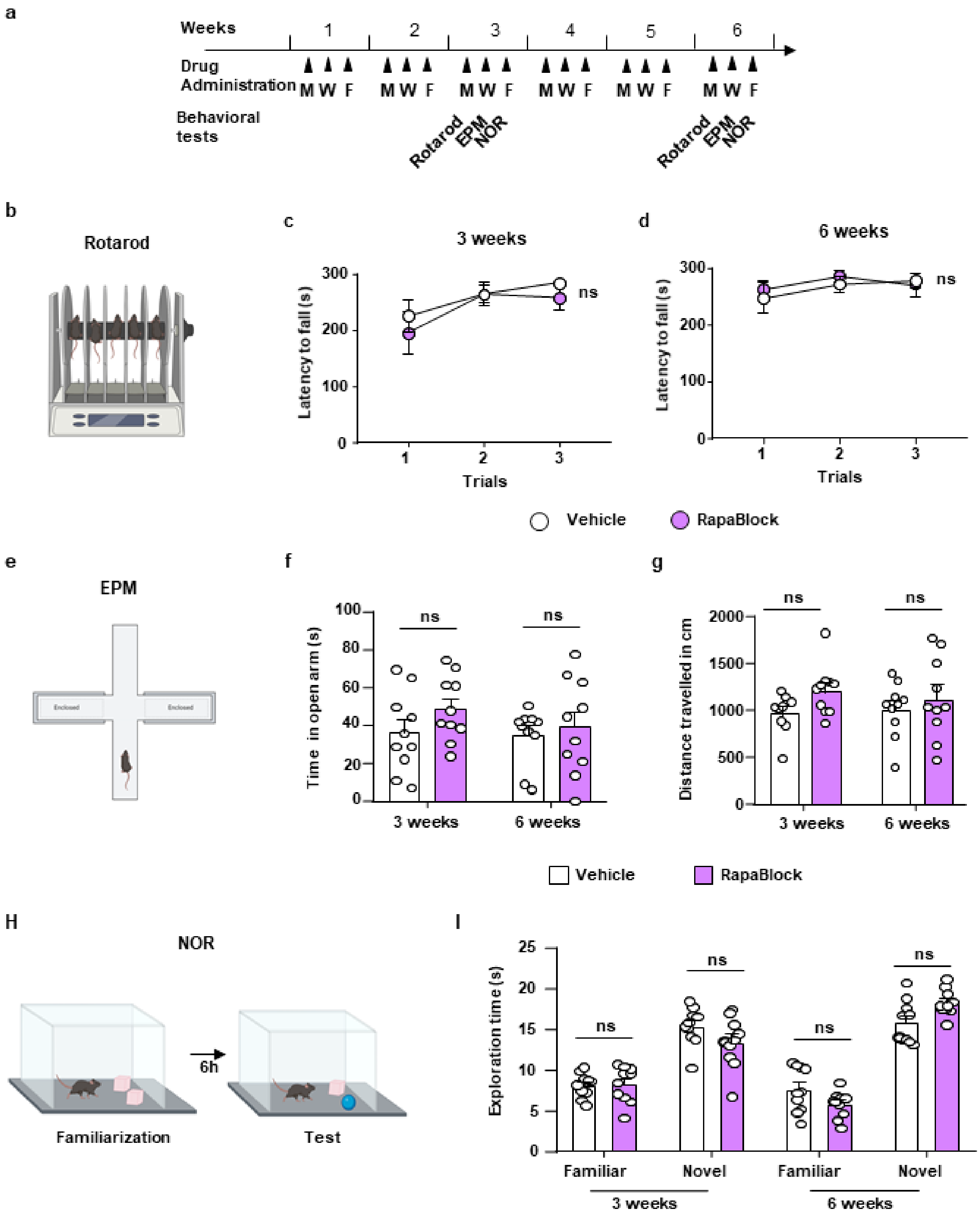

a
Weeks
1
2
3
4
5
6
Drug Administration
M W F
Behavioral tests
Rotarod
EPM
NOR
b
Rotarod
c
3 weeks
Latency to fall (s)
Trials
ns
d
6 weeks
ns
Vehicle
RapaBlock
e
EPM
f
Time in open arm (s)
3 weeks
6 weeks
g
Distance travelled in cm
H
NOR
6h
Familiarization
Test
I
Exploration time (s)
Familiar
Novel

Supplementary Fig. 3. Chronic administration of RapaBlock does not produce adverse behavioral effects. (a) Timeline of experiments. Mice were treated with vehicle (white) or RapaBlock (40mg/kg, pink) 3 times a week for 6 weeks and behavioral tests were conducted at 3 and 6 week. Rotarod test (b), elevated plus maze test (EPM) (e) and Novel object recognition test (NOR) test (h). (c,d) RapaBlock does not alter rotarod performance (Two-way ANOVA: no effect of treatment after 3 weeks ($F1,18 = 0.6013$, $p = 0.4482$) and 6 weeks ($F1,18 = 0.1274$, $p = 0.7253$). (f,g) Time spent in the open arm of the EPM (RM Two-way ANOVA: $F1,18 = 2.435$, $p = 0.1361$) and total distance travelled during the test (RM Two-way ANOVA: $F1,18 = 4.183$, $p = 0.0557$) were not affected by RapaBlock. (i) Time exploring a familiar and novel object were not affected by RapaBlock treatment at both time points (RM Two-way ANOVA: $F1,18 = 0.7542$, $p = 0.3966$). Data are presented as individual data points and mean ± SEM, n=10 per condition. Significance was determined using RM Two-way ANOVA followed by Tukey's multiple comparisons test. ns = non-significant.

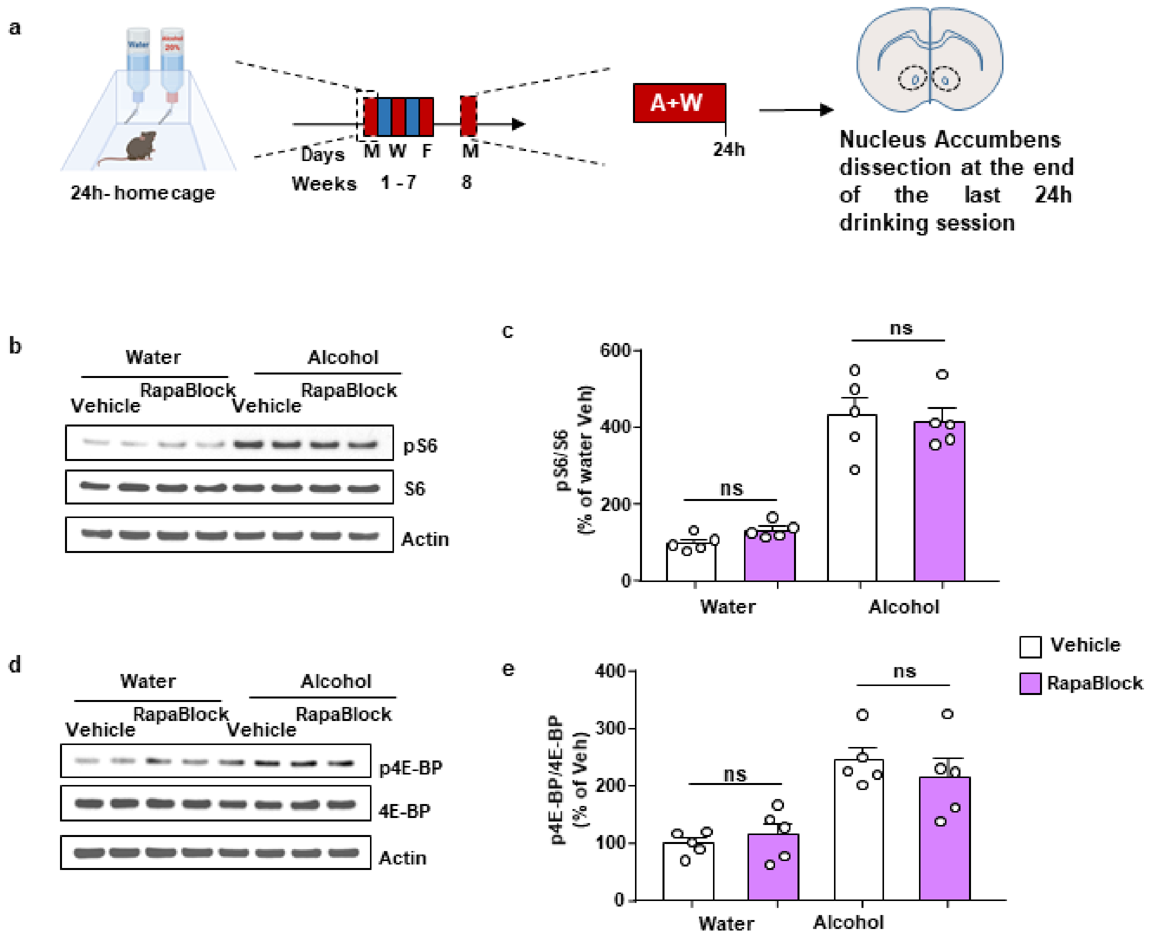

Supplementary Fig. 4. RapaBlock does not alter alcohol-dependent activation of mTORC1 in the Nucleus Accumbens. (a) Timeline of experiment. Mice underwent 7 weeks of IA20%2BC. On week 8, mice received a systemic administration of vehicle (white) or RapaBlock (40mg/kg, pink) 3 hours before the beginning the session, and the NAc was removed at the end of the 24 hours drinking session. (b,d) Representative images of phospho-S6 (pS6) (b) and phospho-4E-BP (p-4E-BP) (d) (top panels), total protein levels of S6 (b) and 4E-BP (d) (middle panels), and actin (bottom panels). (c,e) Data are presented as the individual data points and mean densitometry values of the phosphorylated protein divided by the densitometry values of the total protein ± SEM and expressed as % of vehicle. Significance was determined using Two-way ANOVA. Administration of RapaBlock does not affect the levels of pS6 (Two-way ANOVA: main effect of alcohol ($F1,16=270.2$, $p < 0.0001$), no effect of treatment ($F1,16 = 0.1542$, $p = 0.6997$)). Administration of RapaBlock does not affect the levels of p4E-BP (Two-way ANOVA: main effect of alcohol

($F_{1,16} = 37.31$, $p < 0.0001$), no effect of treatment ($F_{1,16} = 0.1707$, $p = 0.6849$)). n=5 per condition. ns = non-significant.

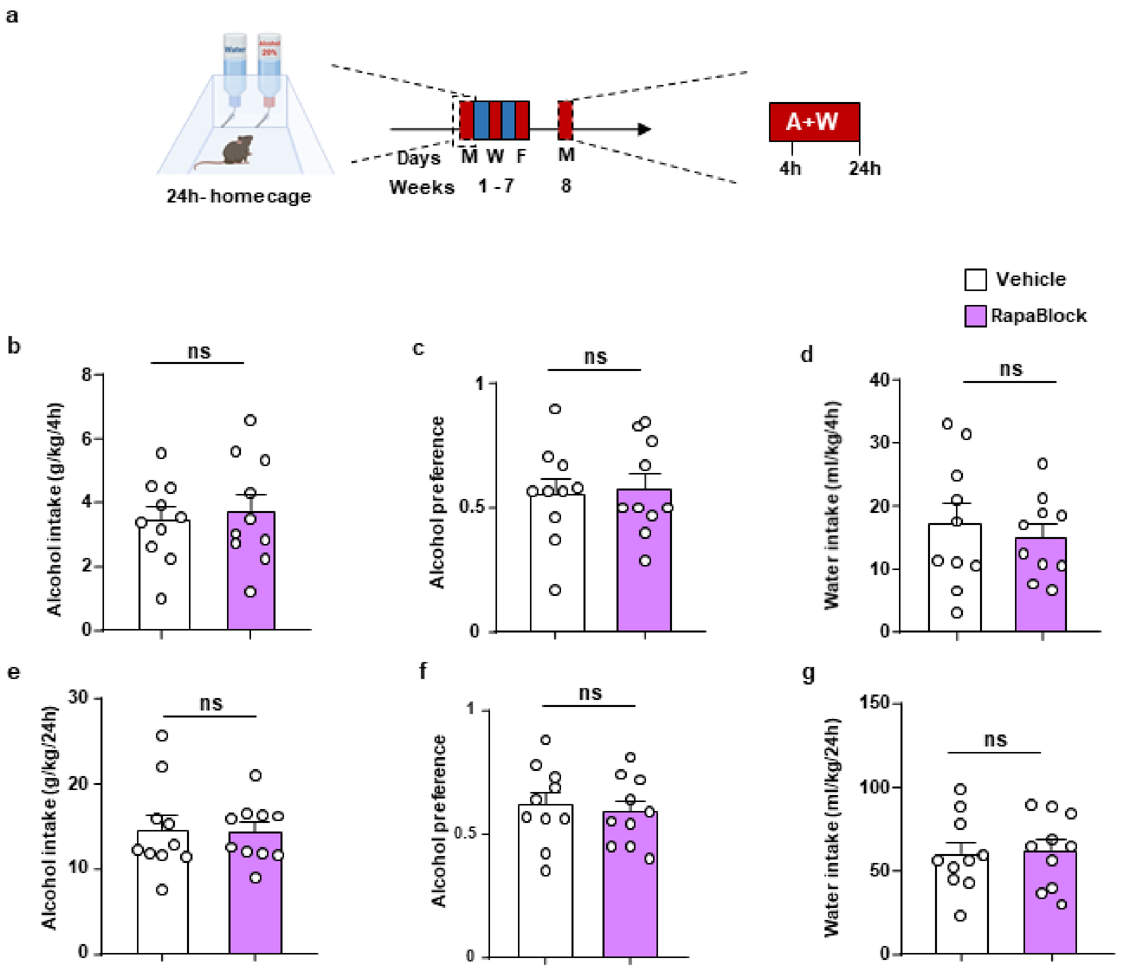

Supplementary Fig. 5. RapaBlock does not alter alcohol intake. (a) Mice underwent 7 weeks of IA20%2BC. On week 8, mice received a systemic administration of vehicle (white) or RapaBlock (40mg/kg, pink) 3 hours before the beginning of drinking session. Alcohol and water intake were measured 4 hours (b, d) and 24 hours later (e, g). Alcohol preference was calculated as the ratio of alcohol intake to total fluid intake at the 4-hour (c) and 24-hour (f) time points. (b-d) Administration of RapaBlock does not alter alcohol intake (Two-tailed unpaired t-test: $t = 0.4256$, $p = 0.6754$, $r^2 = 0.009965$), alcohol preference (Two tailed unpaired t-test: $t = 0.3636$, $p = 0.7207$, $r^2 = 0.007716$) and water intake (Two-tailed unpaired t-test: $t = 0.4192$, $p = 0.6803$, $r^2 = 0.01023$) at the end of the 4-hour session. (e-g) Administration of RapaBlock does not alter alcohol intake (Two-tailed unpaired t-test: $t = 0.1546$, $p = 0.8788$, $r^2 = 0.001327$), alcohol preference (Two-tailed unpaired t-test: $t = 0.1191$, $P = 0.9066$, $r^2 = 0.0008341$) and water intake (Two-tailed unpaired t-test: $t = 0.2297$, $p = 0.8209$, $r^2 = 0.002922$) at the end of the 24-hour session. (b-g) Data are

presented as individual data points and mean ± SEM. Significance was determined using Two-tailed unpaired t-test. n=10 per condition. ns = non-significant.

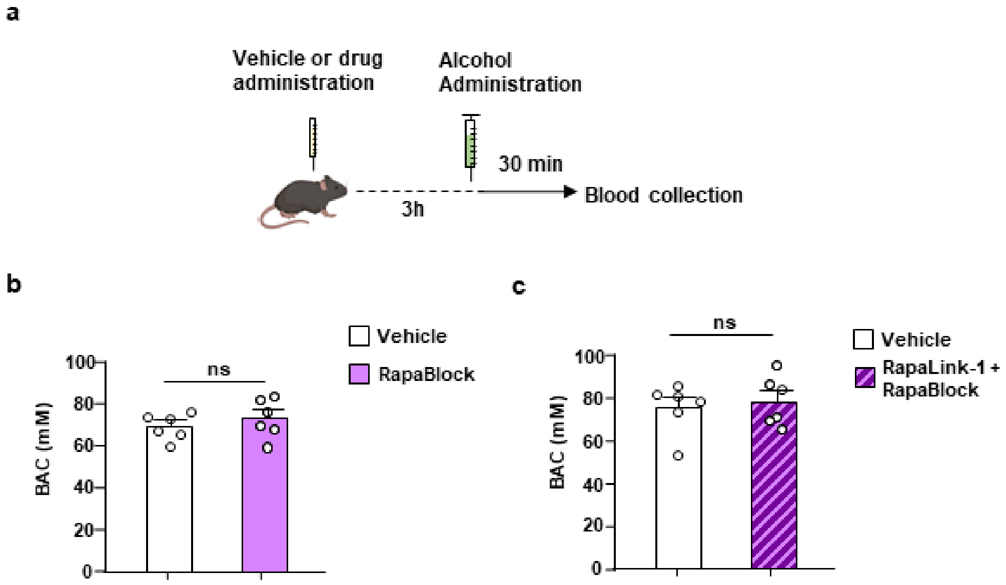

Supplementary Fig. 6. Systemic administration of RapaBlock or a combination of RapaLink-1 and RapaBlock does not alter blood alcohol concentration (BAC). (a) Timeline of experiment. Mice received a systemic administration of vehicle, RapaBlock alone (40 mg/kg, pink) (b) or RapaLink-1 (1mg/kg, purple)+RapaBlock (40 mg/kg, pink) (c). Three hour later, mice received a systemic administration of alcohol (2 g/kg), and BAC was measured 30 minutes later. (b-c) Data are presented as mean ± SEM. (b) BAC is similar in vehicle-treated vs. RapaBlock-treated mice (Two-tailed unpaired t-test: t = 0.8537, p = 0.4133, r^2 =0.06793). (c) BAC is similar in vehicle-treated vs. RapaLink-1+RapaBlock-treated mice (Two-tailed unpaired t-test: t=0.4580, p = 0.6567, r^2 = 0.02055). n = 6 per group. ns = non-significant.

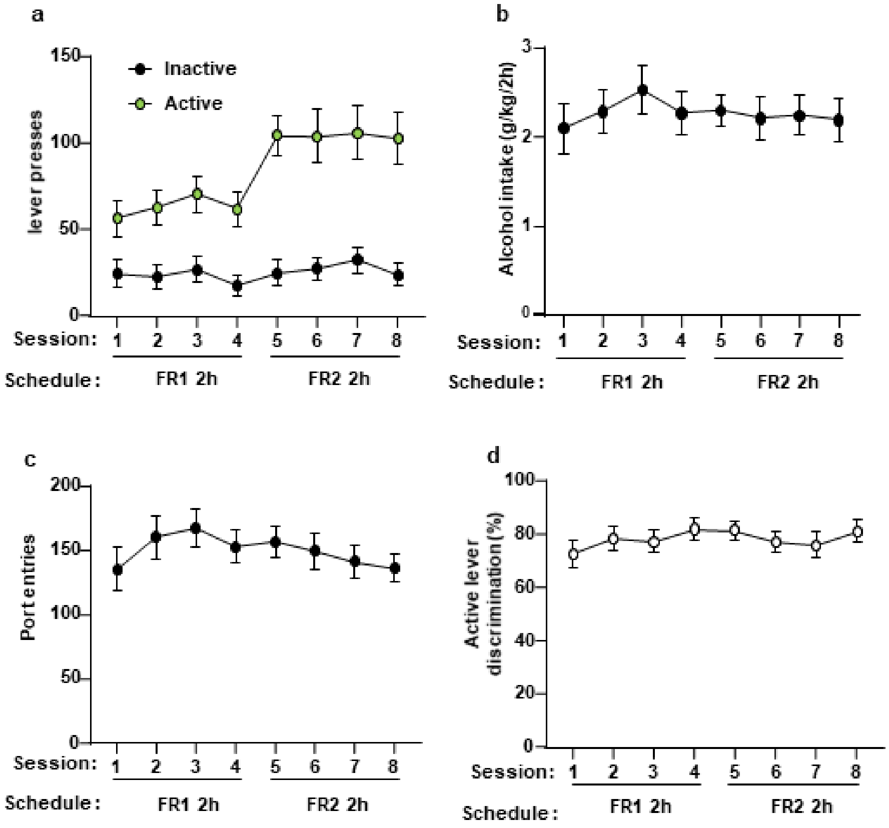

Supplementary Fig. 7. Operant alcohol self-administration training. Mice that underwent IA20%2BC for 7 weeks were trained to self-administer alcohol in operant chambers. (a-c) Number of active (green) and inactive (black) lever presses (a), alcohol consumed (g/kg/2hr) (b), and port entries (c) during alcohol self-administration training period under fixed ratio 1 (FR1) and 2 (FR2). (d) Active lever discrimination is calculated as the percentage ratio of active lever presses versus the total number of lever presses (active + inactive). Data are presented as mean ± SEM. n = 15.

References

1. Rodrik-Outmezguine VS, *et al.* Overcoming mTOR resistance mutations with a new-generation mTOR inhibitor. *Nature* **534**, 272-276 (2016).

2. Zhang Z, Fan Q, Luo X, Lou KJ, Weiss WA, M. SK. Achieving Brain-Restricated mTOR Inhibiton with Binary Pharmacology. *bioRxiv*, doi: https://doi.org/10.1101/2020.1110.1112.336677 (2020).

Supplementary Table S1

Antibodies	Vendor	Catalog #	Lot #	Dilution
phospho-S6 (S235/236)	Cell Signaling	2211	23	1:500
S6	Cell Signaling	2217	7	1:1000
phospho-4E-BP (T37/46)	Cell Signaling	2855	26	1:500
4E-BP	Cell Signaling	9452	5	1:1000
phospho-STAT3 (Y70)	Cell Signaling	9145	43	1:500
STAT3	Cell Signaling	4904	7	1:500
Actin	Sigma Aldrich	A2228	127M4866V	1:10000

Supplementary Table S2: Sequence of primers

Gene		Sequence
Timp1	Forward	5′-GGT GTG CAC AGT GTT TCC CTG TTT-3′
	Reverse	5′-TCC GTC CAC AAA CAG TGA GTG TCA-3’
Col4a1	Forward	5’-CCA TGG TCA GGA CTT GGG TA-3′
	Reverse	5′-AAG GGC ATG GTG CTG AAC T-3′
Gapdh	Forward	5′-CGA CTT CAA CAG CAA CTC CCA CTC TTC C-3′
	Reverse	5′-TGG GTG GTC CAG GGT TTC TTA CTC CTT-3′

Full scans of western blots

Figure 2

For pS6 and S6, set 1 is displayed in the main figure.

For p4EBP and 4EBP, set 2 is displayed in the main figure.

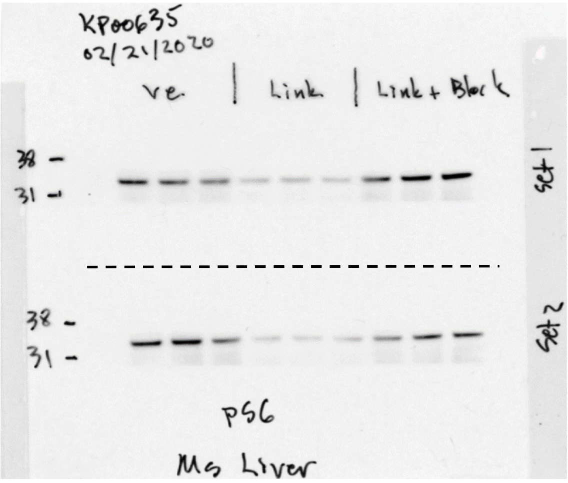

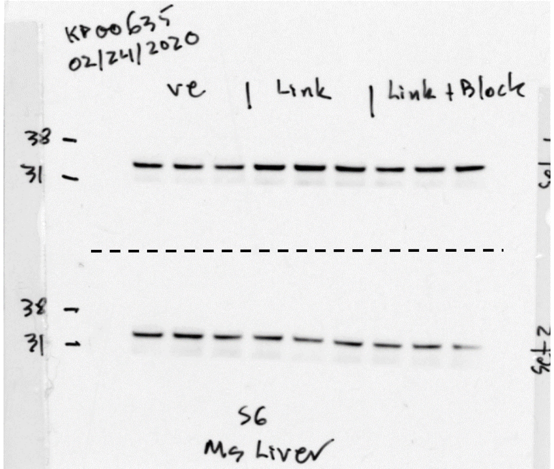

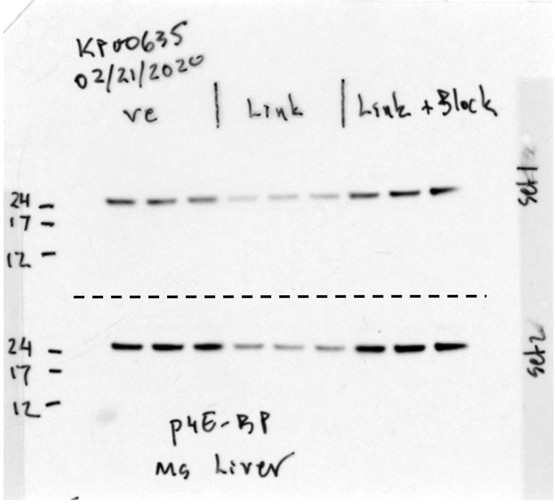

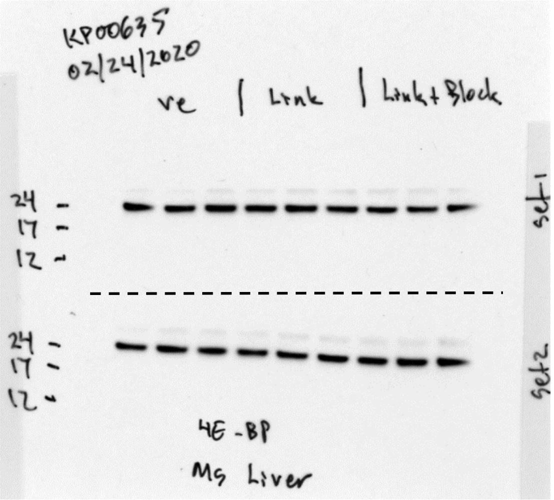

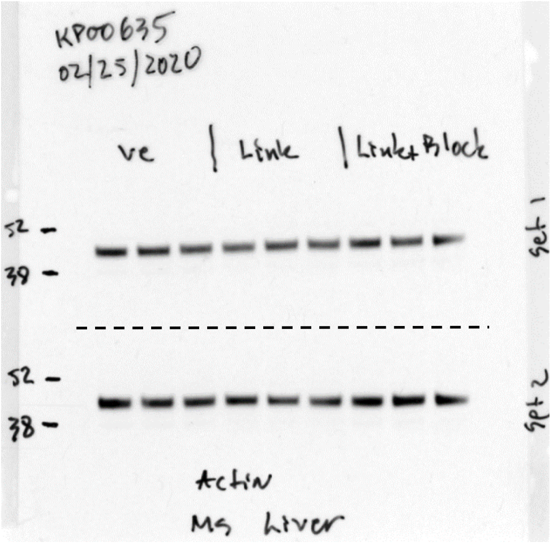

Figure 3

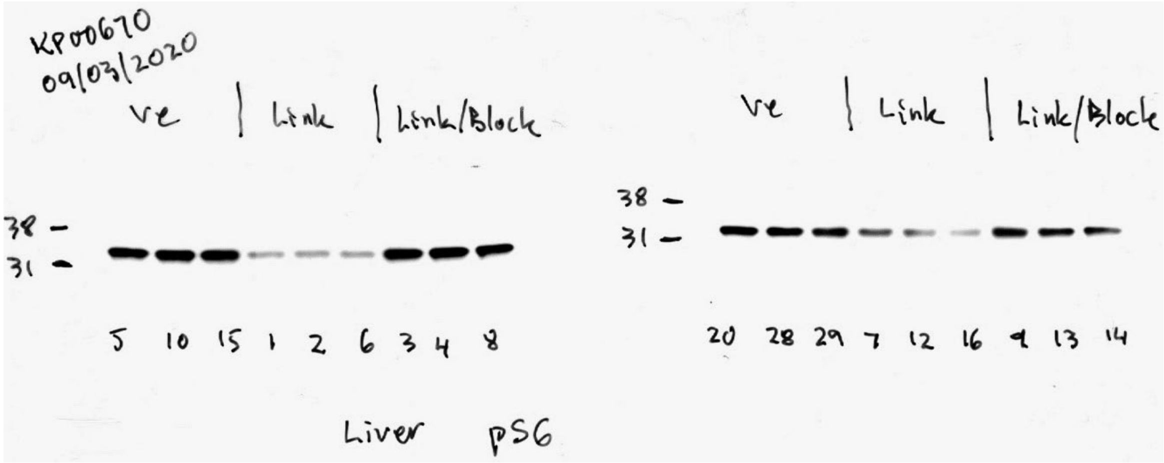
KP00670
09/03/2020
Ve | Link | Link/Block
Ve | Link | Link/Block
38
31
38
31
5 10 15 1 2 6 3 4 8
20 28 29 7 12 16 9 13 14
Liver pS6

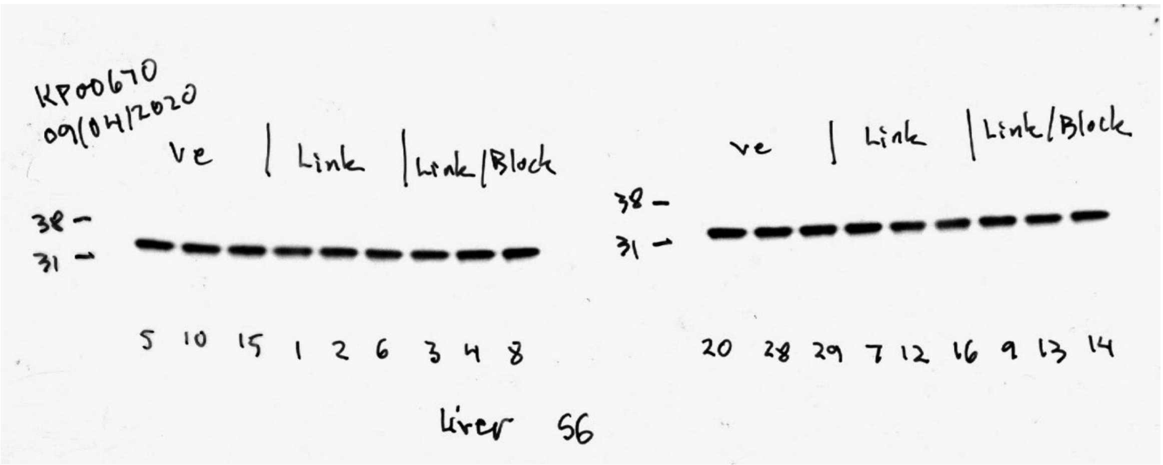
KP00670
09/04/2020
Ve | Link | Link/Block
Ve | Link | Link/Block
38
31
38
31
5 10 15 1 2 6 3 4 8
20 28 29 7 12 16 9 13 14
Liver S6

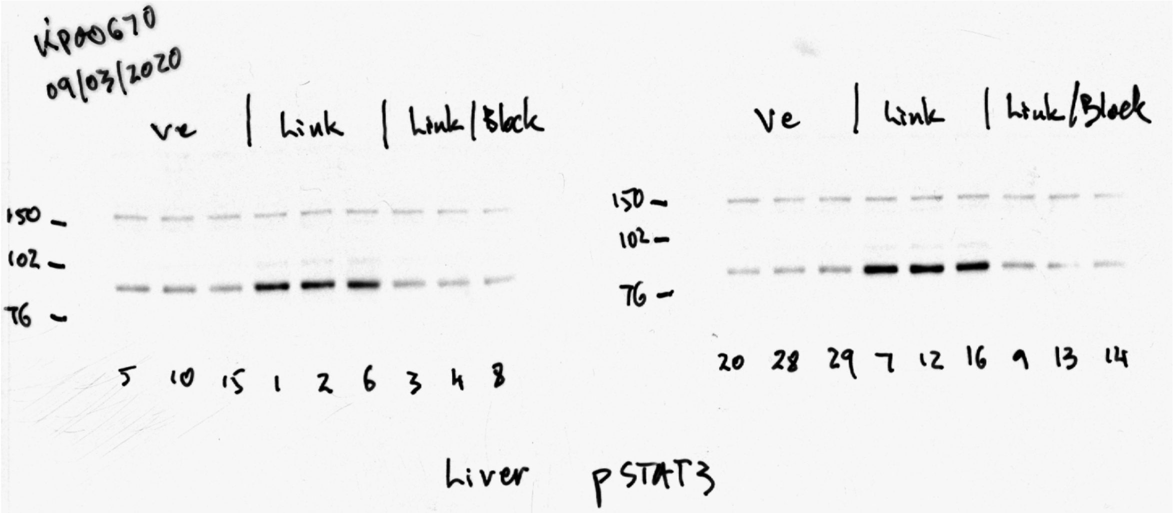
KP00670
09/03/2020
Ve | Link | Link/Block
Ve | Link | Link/Block
150
102
76
150
102
76
5 10 15 1 2 6 3 4 8
20 28 29 7 12 16 9 13 14
Liver pSTAT3

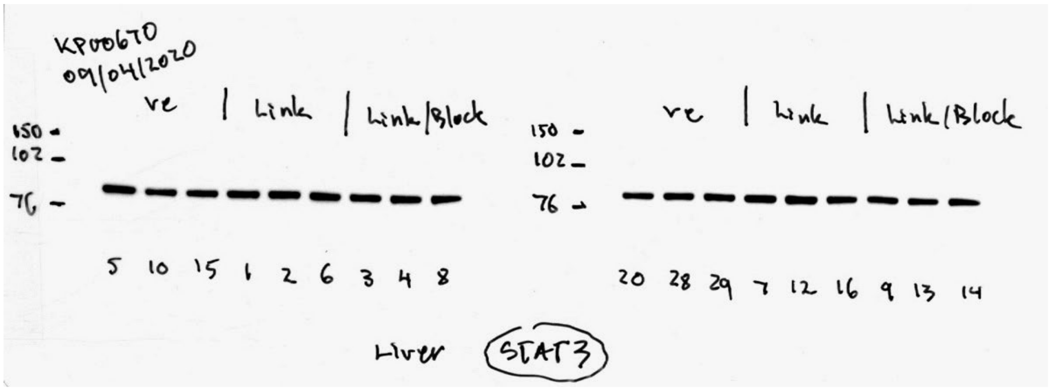

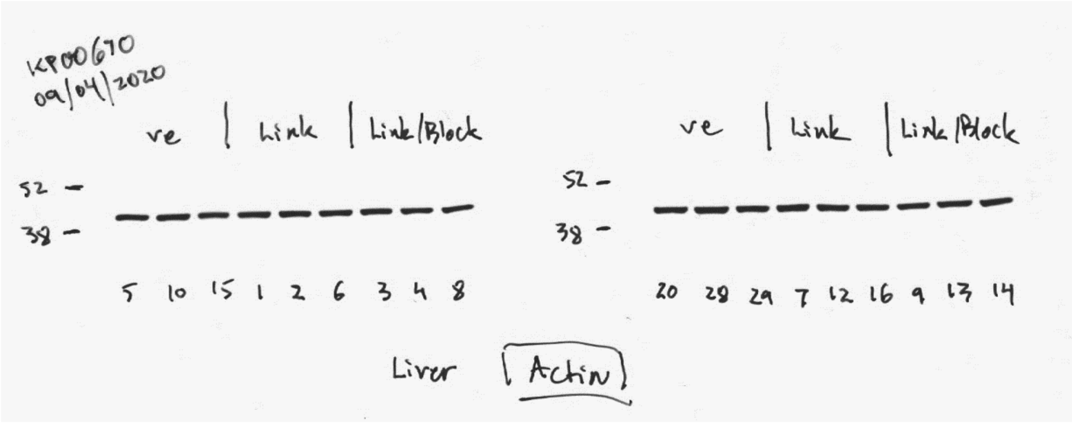

Figure 4

For pS6 and S6, set 3 is displayed in the main figure.

For p4EBP and 4EBP, set 2 is displayed in the main figure.

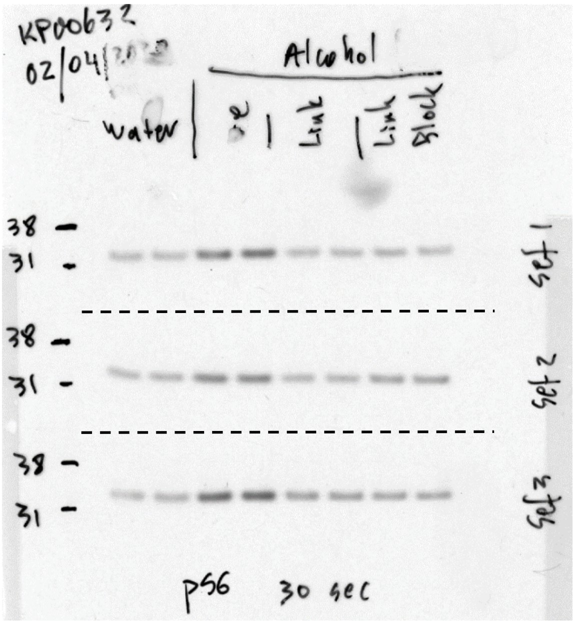

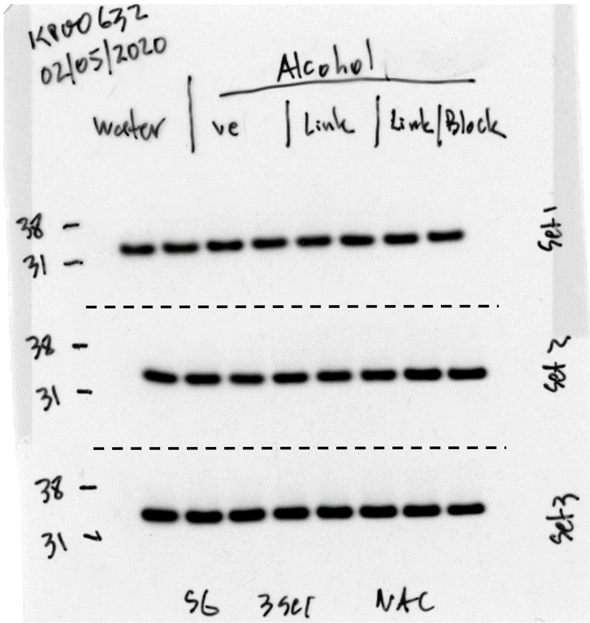

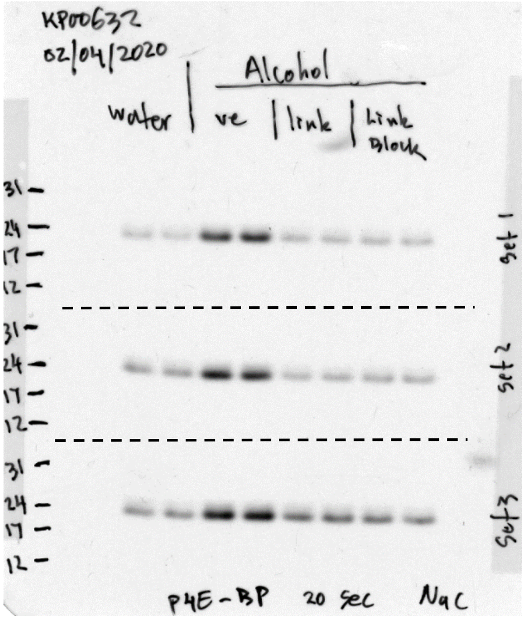
KP00632
02/04/2020
Alcohol
water
ve
link
Link Block
31
24
17
12
set 1
set 2
set 3
P4E-BP
20 sec
NaC

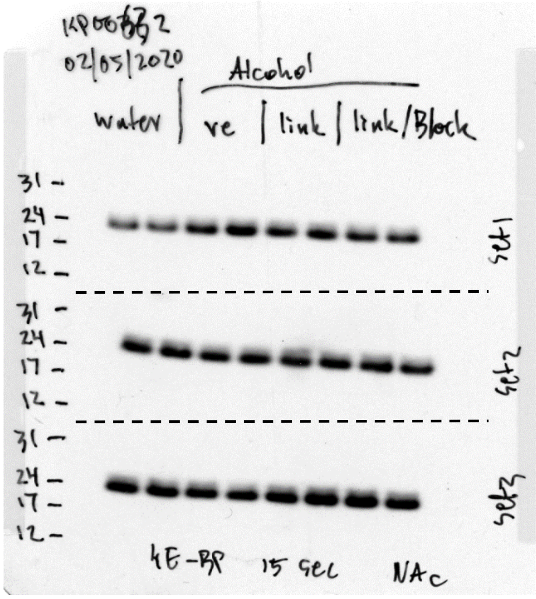
KP00632
02/05/2020
Alcohol
water
ve
link
link/Block
31
24
17
12
set 1
set 2
set 3
4E-BP
15 sec
NAc

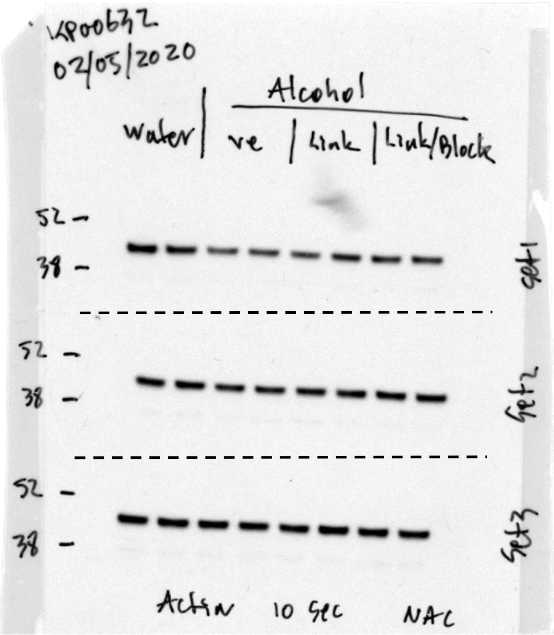
KP00632
02/05/2020
Alcohol
water
ve
Link
Link/Block
52
38
set 1
set 2
set 3
Actin
10 sec
NAC

Supplementary Fig. 2

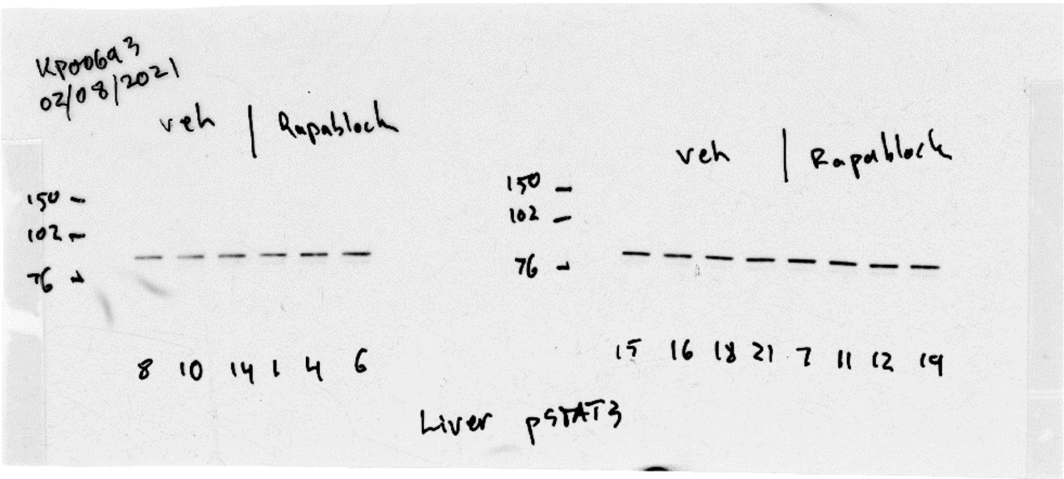

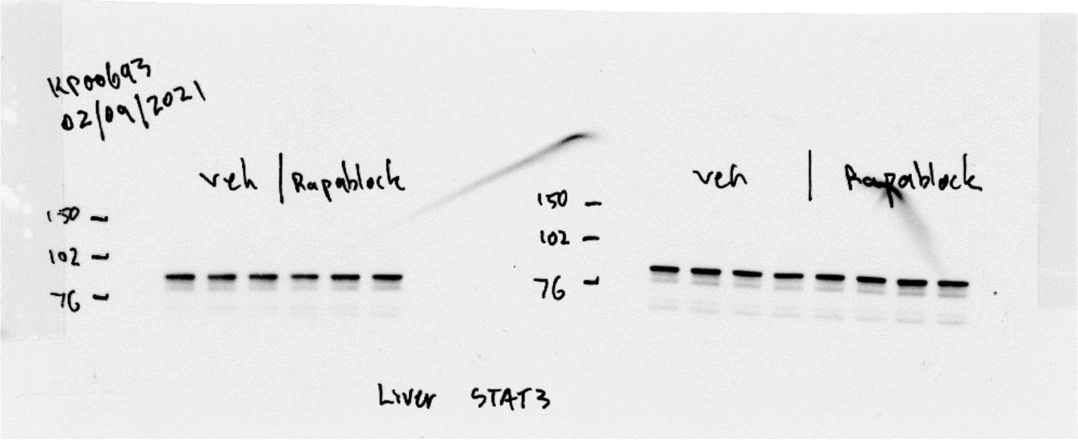

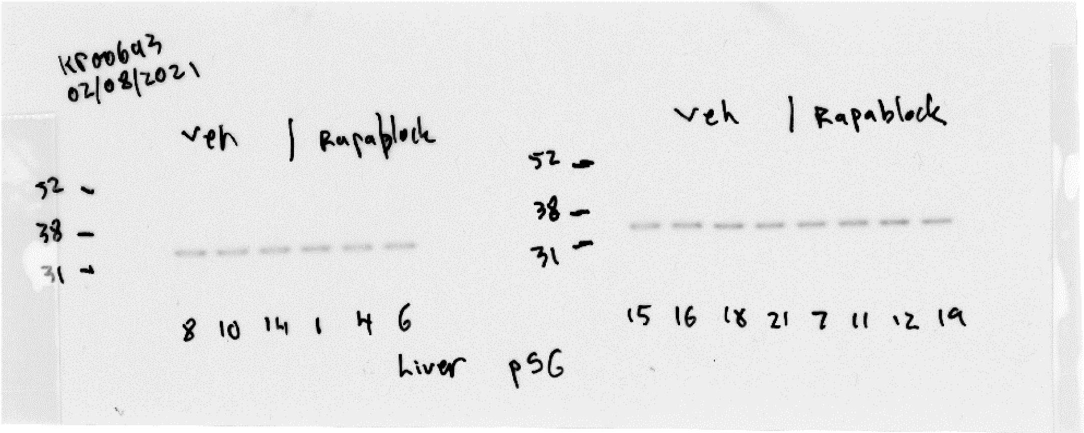

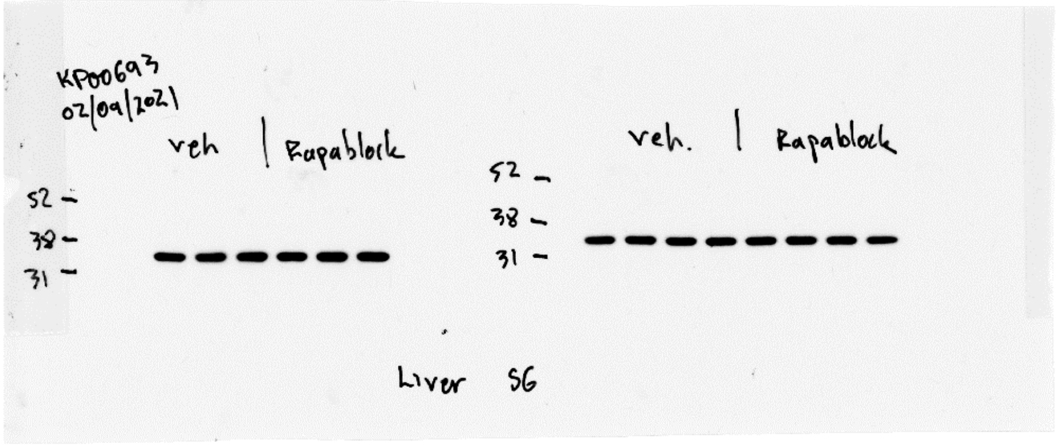

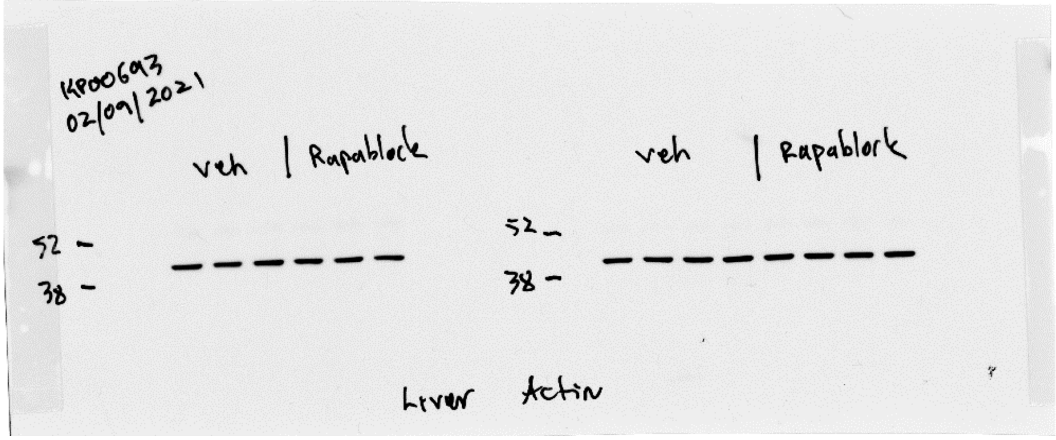

Supplementary Fig. 4

For pS6 and S6, set3 is displayed in the main figure.

For p4EBP and 4EBP, set 1 is displayed in the main figure.

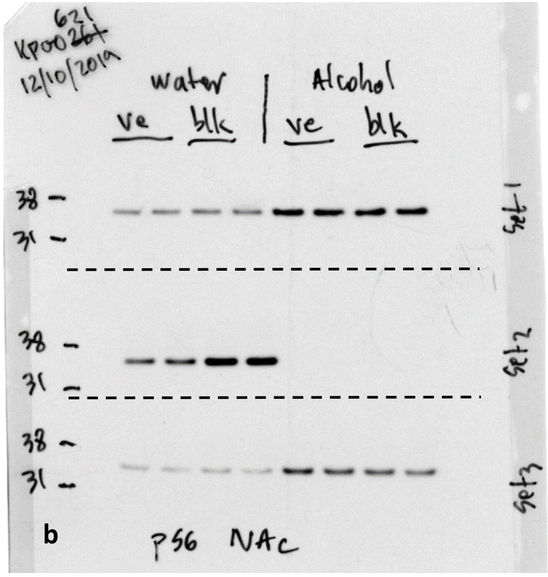

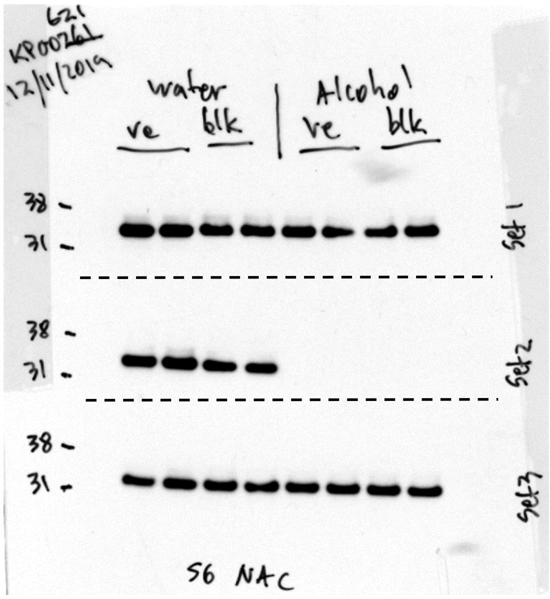

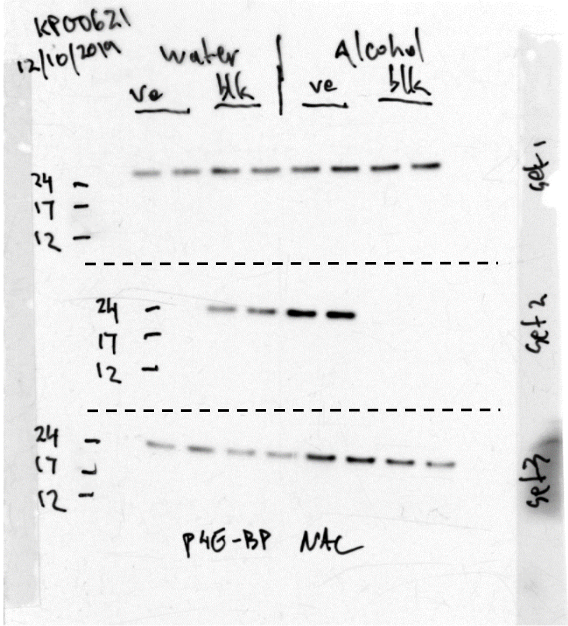
KP00621
12/10/2019
water
Alcohol
ve
blk
ve
blk
24
17
12
set 1
24
17
12
set 2
24
17
12
set 3
P4E-BP NAc

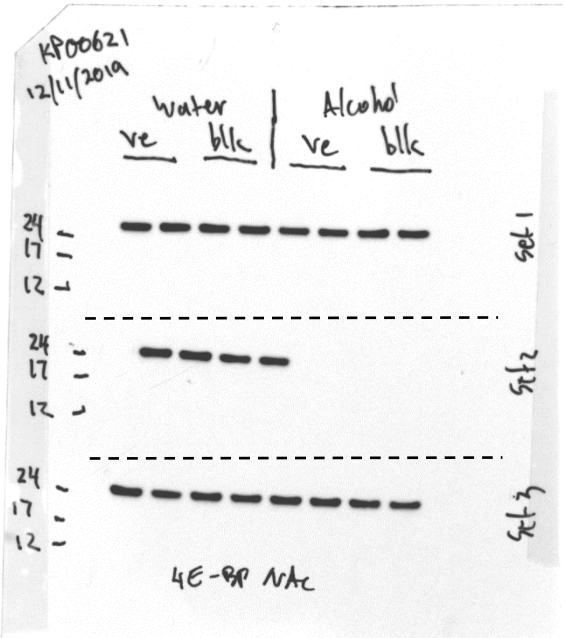
KP00621
12/11/2019
water
Alcohol
ve
blk
ve
blk
24
17
12
set 1
24
17
12
set 2
24
17
12
set 3
4E-BP NAc

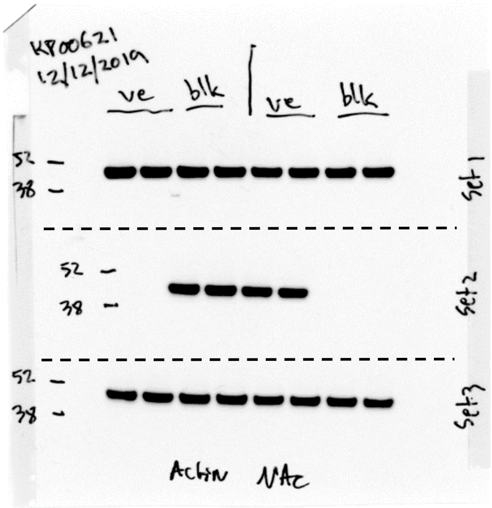
KP00621
12/12/2019
ve
blk
ve
blk
52
38
set 1
52
38
set 2
52
38
set 3
Actin NAc
