# Supplementary material for: Brain-specific inhibition of mTORC1 eliminates side effects resulting from mTORC1 blockade in the periphery and reduces alcohol intake in mice
Source: Nat Commun. 2021 Jul 27;12:4407. doi: 10.1038/s41467-021-24567-x (PMC8316332; doi:10.1038/s41467-021-24567-x)
Supplement: Supplementary file 3 — Reporting Summary [file 41467_2021_24567_MOESM3_ESM.pdf]

nature research

Corresponding author(s): Dorit Ron

Last updated by author(s): May 17, 2021

Reporting Summary

Nature Research wishes to improve the reproducibility of the work that we publish. This form provides structure for consistency and transparency in reporting. For further information on Nature Research policies, see our Editorial Policies and the Editorial Policy Checklist.

Statistics

For all statistical analyses, confirm that the following items are present in the figure legend, table legend, main text, or Methods section.

n/a	Confirmed	
☐	☒	The exact sample size (*n*) for each experimental group/condition, given as a discrete number and unit of measurement
☐	☒	A statement on whether measurements were taken from distinct samples or whether the same sample was measured repeatedly
☐	☒	The statistical test(s) used AND whether they are one- or two-sided<br>*Only common tests should be described solely by name; describe more complex techniques in the Methods section.*
☒	☐	A description of all covariates tested
☐	☒	A description of any assumptions or corrections, such as tests of normality and adjustment for multiple comparisons
☐	☒	A full description of the statistical parameters including central tendency (e.g. means) or other basic estimates (e.g. regression coefficient) AND variation (e.g. standard deviation) or associated estimates of uncertainty (e.g. confidence intervals)
☐	☒	For null hypothesis testing, the test statistic (e.g. *F*, *t*, *r*) with confidence intervals, effect sizes, degrees of freedom and *P* value noted<br>*Give P values as exact values whenever suitable.*
☒	☐	For Bayesian analysis, information on the choice of priors and Markov chain Monte Carlo settings
☒	☐	For hierarchical and complex designs, identification of the appropriate level for tests and full reporting of outcomes
☐	☒	Estimates of effect sizes (e.g. Cohen's *d*, Pearson's *r*), indicating how they were calculated

Our web collection on statistics for biologists contains articles on many of the points above.

Software and code

Policy information about availability of computer code

Data collection	PRISM software (Graphpad, version 8); Excel (Microsoft)
Data analysis	PRISM software (Graphpad, version 8)

For manuscripts utilizing custom algorithms or software that are central to the research but not yet described in published literature, software must be made available to editors and reviewers. We strongly encourage code deposition in a community repository (e.g. GitHub). See the Nature Research guidelines for submitting code & software for further information.

Data

Policy information about availability of data

All manuscripts must include a data availability statement. This statement should provide the following information, where applicable:

- Accession codes, unique identifiers, or web links for publicly available datasets
- A list of figures that have associated raw data
- A description of any restrictions on data availability

Raw data are available in the Supplementary Information file. Source data are also provided with the paper.

Field-specific reporting

Life sciences study design

All studies must disclose on these points even when the disclosure is negative.

Sample size	No statistical methods were explicitly used for predetermination of sample size. Sample sizes were similar those used in previous publications (Laguesse et al. Neuron, 2017, Morisot et al. Addiction Biology , 2018, Laguesse et al. Neuropsychopharmacology, 2018).
Data exclusions	No data were excluded
Replication	All experiments had biological replicates with similar results. We included detailed numbers in the manuscript.
Randomization	Mice were randomly assigned to the treatment groups.
Blinding	All the behavioral experiments were performed blinded to the conditions. All tests were requiring monitoring by a camera were analyzed by blinded experimenter. The biochemical experiments were not performed blindly.

Reporting for specific materials, systems and methods

We require information from authors about some types of materials, experimental systems and methods used in many studies. Here, indicate whether each material, system or method listed is relevant to your study. If you are not sure if a list item applies to your research, read the appropriate section before selecting a response.

Materials & experimental systems

n/a	Involved in the study	
☐	☒	Antibodies
☒	☐	Eukaryotic cell lines
☒	☐	Palaeontology and archaeology
☐	☒	Animals and other organisms
☒	☐	Human research participants
☒	☐	Clinical data
☒	☐	Dual use research of concern

Methods

n/a	Involved in the study	
☒	☐	ChIP-seq
☒	☐	Flow cytometry
☒	☐	MRI-based neuroimaging

Antibodies

Antibodies used	Anti-phospho-S6 (S235/236, 1:500), anti-S6 (1:1000), anti-phospho-4E-BP (T37/46, 1:500), anti-4E-BP (1:1000), anti-phosphoTyr705-STAT3 (1:500) and anti-STAT3 (1:500) antibodies were purchased from Cell Signaling Technology (Danvers, MA). Anti-Actin (1:10,000) antibodies was purchased from Sigma Aldrich (St. Louis, MO). Anti Rabbit Horseradish Peroxidase and anti Mouse Horseradish Peroxidase were purchased from Jackson ImmunoResearch. Catalogue number, lot number and dilution used are listed in Supplementary Table 1.
Validation	All antibodies were purchased from qualified vendors that provide validation on the website of the manufacturer.<br>We validated each of the antibodies in house and made sure that the band of the protein is at the right molecular weight.<br>All antibodies utilized in the study are routinely used. For example, anti--phospho-S6, anti-S6, anti-phospho-4E-BP, anti-4E-BP (Barak et al. Nature Neuroscience 2013, Laguesse et al. Addiction Biology 2016, Rodrik-Outmezguine et al. Nature 2016, Bao et al. Nat Commun 11, 5878, 2020), anti-phospho-STAT, STAT antibodies (Atsushi U et al. Cell Metabolism 2014).

Animals and other organisms

Policy information about studies involving animals; ARRIVE guidelines recommended for reporting animal research

Laboratory animals	Male C57BL/6J mice (Jackson Laboratory, Bar Harbor, ME) were 6-7 weeks old at the beginning of the experiment. Mice were individually housed in separate temperature- and humidity- controlled rooms (temperature and humidity were kept constant at 22 ± 2°C, and relative humidity was maintained at 50 ± 5%) under a 12-hour light/dark cycle (lights on at 07:00 AM) or a reversed 12 hour light/dark cycle (lights on at 10:00 PM) with food and water available ad libitum.
Wild animals	The study did not include wild animals.
Field-collected samples	The study did not include samples collected from the field.
Ethics oversight	All animal procedures were approved by the University of California San Francisco (UCSF) Institutional Animal Care and Use Committee and conducted in agreement with the Association for Assessment and Accreditation of Laboratory Animal Care (AAALAC, UCSF).

Note that full information on the approval of the study protocol must also be provided in the manuscript.
